# New Trend of Physical Activity and Exercise for Health Promotion and Functional Ability

**DOI:** 10.3390/ijerph19137939

**Published:** 2022-06-28

**Authors:** Wonil Park, Hun-Young Park

**Affiliations:** 1 Industry Academic Cooperation Foundation, Chung-Ang University, 84 Heukseok-ro, Dongjak-gu, Seoul 06974, Korea; wonilpark01@cau.ac.kr; 2 Physical Activity and Performance Institute (PAPI), Konkuk University, 120 Neungdong-ro, Gwangjin-gu, Seoul 05029, Korea; 3 Department of Sports Medicine and Science, Graduate School, Konkuk University, 120 Neungdong-ro, Gwangjin-gu, Seoul 05029, Korea

## 1. Introduction

Regular physical activity and exercise can improve your health and reduce your risk of developing various diseases including type 2 diabetes, cardiovascular disease, and cancer. Physical activity and exercise can have numerous immediate and chronic health benefits, such as managing weight, blood cholesterol level, blood pressure, and muscles [1].

Most importantly, regular physical activity and exercise can offer a better quality of life (e.g., more energy, a better mood, feeling more relaxed, and sleeping better). The American College of Sports Medicine (ACSM) provides recommendations and guidelines for physical activity and exercise that all healthy adults aged 18–65 years should participate in moderate-intensity aerobic activity for a minimum of 30 min five days per week or vigorous-intensity aerobic activity for a minimum of 20 min three days per week. In addition, adults should maintain or increase muscular strength and endurance for a minimum of two days per week (ACSM’s Guidelines for Exercise Testing and Prescription) [2].

Taken together, both aerobic and strength activities are important for optimal physical fitness. While practical concerns such as busy schedules and poor health can make exercise more challenging, for most of us, the biggest barriers are mental. Maybe it is a lack of confidence that keeps us from taking positive steps, or it is that the motivation easily burns out, or that we are too quickly discouraged and give up. Therefore, new exercise methods that enable exercise to be sustainable for managing health are essential. Among the many new exercise trends, we would like to briefly introduce some of the exercise modalities including blood flow restriction, electromyostimulation, hypoxic training, vibration, and interval training.

## 2. Blood Flow Restriction

Blood flow restriction training is an effective exercise method developed by Dr. Sato Yoshiyaki of Japan in 1966 that uses a blood flow control band to increase growth hormone secretion and improve blood circulation and metabolism to prevent and improve adult diseases, shorten rehabilitation periods, and enhance cardiovascular function. The purpose of blood flow restriction training is to temporarily restrict venous blood flow to the peripheral area of the extremity through pressurization and to improve blood flow through exercise after pressurization [3]. In general, strength training has guidelines such as intensity, frequency, shape, set, and number of repetitions of exercise [2]. In traditional training, exercise with a load of more than 65% of 1 RM can be expected, but in blood flow restriction training programs, a 20 to 50% level of 1 RM is maintained, and 1 set is performed between 25 and 30 times [4]. In the case of this training, the upper and lower body should be recessed for 15 s and 30 s, respectively. Moreover, the frequency of daytime exercise is not as tiring as traditional muscle exercise, and recovery is faster, so pressure can be adjusted in six steps depending on the exercise’s characteristics or purpose [3]. Blood flow restriction training was conducted twice a day at 20% of 1RM for 2 weeks, showing an effect of increasing the quadriceps muscles’ cross-sectional area by 7.8% and the muscle fiber’s cross-sectional area by 5.9% for type 1 and 27.6% in type 2 [5]. In addition, blood restriction training can lead to aerobic to anaerobic metabolic transition in low-intensity exercise due to blood flow restrictions and congestion, which leads to the strengthening of the hypoxic environment and muscle nerve activity and activation of the endocrine system. When blood flow is restricted, oxygen supply to the muscles is not smooth, and removal of metabolites such as lactic acid is hindered. As a result, exercise units including multiple fast muscle fibers are mobilized to maintain muscle strength [6]. Muscle training is also different from conventional resistance exercise in that muscle training is performed in the order of muscle fibers and depending on strength [5]. As such, low-intensity blood flow restriction can have the same effect as high-intensity resistance exercise, and it is also effective as a rehabilitation exercise for elderly or sick people with sarcopenia [7,8]. However, blood flow restriction training is not always effective. The side effects during blood flow restriction training are still very rare [9]. The most common side effect is subcutaneous bleeding, which occurs temporarily for a short time and decreases with continuous training [6]. In addition, venous thrombosis, rhabdomyolysis, and cerebral anemia symptoms have been reported rarely, but no serious side effects or complications have been caused [9]. There are no cases of death from pressurized training compared to 0 to 2.5% [10] in extreme exercise among older people, so pressurized training is considered a safer and more effective training method for patients with fractures, diabetes, high blood pressure, and cerebrovascular diseases. [11].

## 3. Electromyostimulation

Electromyostimulation training (EMS training) is a principle in which a current stimulates the muscle nerves to cause artificial muscle contraction and is used for muscle strength [12]. In the past, it was mainly used for the improvement and treatment of muscular nervous system dysfunction, the prevention of postoperative muscular atrophy, and pain relief [13], but in 1977, Kots in Russia showed positive effects after applying EMS training to elite athletes. Studies have been conducted on various subjects, including elite athletes and the elderly, as well as healthy adults [14]. In particular, sports medicine is applied to the field for the purpose of strengthening muscle strength, rehabilitation, edema control, and recovery to improve performance through EMS training [15]. The increase in muscle strength caused by the implementation of EMS training can be explained by a mechanism similar to the increase in muscle strength by the high-strength, low-repeated muscle strength protocol performed in general resistance exercise. Seyri & Maffiuletti [16] stated that EMS training significantly improved muscle strength and anaerobic power in healthy adults and increased voluntary muscle activity. Wall et al. [17] reported an increase in muscle synthesis after EMS treatment in older adults with diabetes. However, despite these many advantages and limitations, existing EMS training is passive and limited to local areas, so full-body strength training is limited [18]. Deley & Babault [19] reported improvements in cardiopulmonary ability through EMS training [19]. To compensate for these shortcomings, Whole-body EMS (WB-EMS) training equipment has been developed to perform full-body exercises and has become a popular movement in Europe since the early 2000s, mainly in Germany [20]. WB-EMS training is a new type of training based on the latest electrical stimulation control technology that allows short-time, high-intensity exercise to be performed with less movement, and it is considered a very suitable exercise for people who are not interested in exercise or who are physically challenged [18,21]. WB-EMS training has been reported to increase the protein synthesis of skeletal muscle in older adults in inactive frailty precursors [22]. In particular, WB-EMS has been reported to have a positive effect on the body composition of older people with low physical activity and a high risk of sarcopenia [21]. In a study of cardiovascular variables for WB-EMS, it was reported that mean blood pressure (MAP) decreased by 4.9% and 8.1% in older women after WB-EMS training, significantly improving the cardiovascular system [23].

## 4. Hypoxic Training

Altitude training, also known as hypoxic training, involves exercising in, living in, or otherwise breathing oxygen-reduced air. This is performed to improve athletic performance and physical wellness. Training under a state of hypoxia can also help people acclimatize to higher altitudes. Since the 1968 Mexico Olympics (2240 m) held in Mexico City, many studies have been conducted on altitude training to improve the performance of aerobic elite athletes [24]. In order for elite athletes to achieve successful results in international competitions such as the Olympics and world championships, they require at least six years of athletic experience and at most 16 years of experience. Elite athletes try to improve cardiopulmonary endurance, an important factor in order to achieve good results in competitions. Altitude training is one of the training methods which has been referred to as “altitude”, with elevations of more than 3000 m above sea level, and is relatively flat, primarily with steep cliffs, located above 500 m above sea level from a geographical point of view [25]. Based on this, some elite track and field athletes conducted altitude training in Chamonix, France, Albuquerque in the United States, and Kunming in China [26].

A low-pressure and low-oxygen environment means an environment in which the oxygen partial pressure in the atmosphere is lower than the pressure at sea level (760 torr). Exposure to this low-pressure and low-oxygen environment results in physiological adaptation at the level of blood components that affect the oxygen-carrying capacity of the blood [27], although the degree of adaptation varies depending on the period of exposure. Physiological adaptation due to exposure to high-altitude environments is caused by mechanisms to compensate for the reduction in alveolar oxygen partial pressure (P_A_O_2_), arterial oxygen saturation (%SaO_2_), arterial oxygen content (CaO_2_), and sympathetic oxygen difference (a-vO_2_diff) in the body due to reduced oxygen partial pressure (PO_2_). In addition, aerobic exercise ability is reduced during exercise in such low-pressure and low-oxygen environments [28]. In endurance sports, exercise performance is greatly influenced by oxygen transport capacity from the whole body to local areas such as active muscles (Park et al., 2018). This improvement in oxygen transport capacity results in an increased efficiency of aerobic energy production, resulting in increased maximal oxygen uptake (VO_2_ max), as well as improved time to fatigue during exercise and increased exercise intensity [29]. Training in natural and artificial altitude environments has been reported to improve the performance of aerobic sports by improving their oxygen transport and utilization through hematologic changes such as hemoglobin (Hb) mass increase and red blood cell (RBC) production and cardiovascular function and skeletal muscle oxygen availability. Recently, some researchers have applied a natural artificial notification environment to improve muscle function through resistance training. As suggested by Schoenfeld et al. (2013), this principle is based on the mobilization of muscle fibers with increased metabolic stress due to exercise, increased hormone release, changes in myokine production, increased production of reactive oxygen species, and cell edema to induce muscle hypertrophy and muscle function improvement [30]. In the case of studies related to metabolites, several evidences argue that resistant exercise training performed in a low-oxygen environment can lead to greater stimulation of metabolites to improve muscle hypertrophy and muscle function, but the correlation is not clear [31,32]. Park & Lim [33] showed an improvement in muscle strength and muscular endurance and an increase in the viscous endothelial growth factor (GH, IGF-1, VEGF) through the application of 6 weeks of resistive band exercise training by swimmers in a low-oxygen environment. However, it could not explain the correlation between muscle function and hormones. In particular, the current role of systemic endocrine responses in muscle hypertrophy and muscle function improvement is controversial among many scientists. Therefore, additional mechanical research is needed.

## 5. Whole Body Vibration Training

Traditional training, such as aerobic exercise or resistance exercise, is effective in improving constitution and fitness [34,35] but may be unsuitable for those who have difficulty performing the desired intensity and amount of exercise, especially for obese or elderly people [36]. WBVT refers to a method of stimulating a specific muscle group by vibrating the whole body. It is mainly driven by standing on a vibrating scaffold and can perform a variety of motion movements in situations where vibration is applied without external resistance [37]. These vibrational stimuli (to be precise, amplitude and frequency) mechanically stimulate intrinsic receptors such as the proximal spindle, which can lead to the response and adaptation of the muscular nervous system to enhance muscular endurance and muscle strength [38]. As a result of applying WBVT to obese people, most studies reported improved body composition, muscle strength, blood pressure, and blood variables (TG, LDL-C, insulin, leptin, adiponectin) and improved PWA, PWV, and Aix predicting cardiovascular disease, which were more effective in obese women. However, an accurate protocol has not yet been presented for exercise intensity (amplitude, frequency, exposure time), and it is reported that there will be a difference in effect. Therefore, considering the short research period of prior studies, it was suggested that a longer study would be necessary [36]. The study of applying WBVT to older adults showed a better effect on bone density (especially important femur, hip, and lumbar) than traditional training [39], but conflicting results suggest that more research is needed [40]. Most studies applying WBVT to older adults showed no better muscle strength compared to traditional muscle strength training, but showed improvements compared to controls, and improved functional strength important for preventing falls such as stride, walking speed, reaction speed, and SLR [37].

## 6. Interval Training

Interval training refers to an interval set according to the training intensity (or time, distance) and a training method that exercises with relatively low intensity for the number of intervals desired for recovery. The basic principle of interval training is to exercise at a high exercise intensity during a required distance or exercise time, then exercise at a low exercise intensity for a recovery period, and then exercise again at a high exercise intensity [41]. The purpose of interval training is to train players to familiarize themselves with the high performance required when competing against other players in competitions so that they can perform well during a given exercise time and pace. Interval training usually takes place a few weeks before a race [42]. In the initial stage of interval training, high-intensity exercise for a short period of time and long rest periods should be set. After becoming used to these training methods to some extent, the exercise time increases and the rest time shortens to increase the amount of exercise and interval intensity [43]. Eventually, the focus turns to exercise intensity, and if not, the focus is on exercise distance (e.g., marathon race). The large division of interval training consisting of 1. High-Intensity Interval Training (HIIT) and 2. Sprint Interval Training (SIT” supramaximal”) has a greater effect than traditional medium-intensity exercise on improving mitochondria, which are important in terms of maximum oxygen intake and aerobic function [44]. It has also been reported to be more effective in improving cardiovascular disease, metabolic disease, blood pressure, body composition, and physical strength than traditional medium-intensity exercise, and it has been suggested as a very effective exercise method for the elderly and obese people who are vulnerable to the above risk factors [45,46]. In most studies applying HIIT to the elderly, muscle strength, cardiopulmonary endurance, blood TG, and functional fitness were improved, which can lower the risk of sarcopenia, which should be considered significant in the elderly [45]. This effect was most effectively suggested by repeating 40 min of exercise and 6 sets twice a week for more than 12 weeks and within 60 s of training and 90 s of rest. When HIIT was applied to obese people, it was very effective in reducing waist circumference and body fat, and 10 weeks of HIIT reduced body fat without weight change [46]. In particular, when comparing the effects by applying exercise time such as traditional medium-intensity training, it seems to be suitable for those who lack time. These effects are known to be ineffective when not combined with diet [47], but when comparing moderate-intensity training and HIIT, only HIIT shows a significant decrease in visceral fat, so exercise should be combined to effectively improve abdominal obesity [48].
